# An information-theoretical analysis of gene nucleotide sequence structuredness for a selection of aging and cancer-related genes

**DOI:** 10.5808/GI.2020.18.4.e41

**Published:** 2020-12-08

**Authors:** David Blokh, Joseph Gitarts, Ilia Stambler

**Affiliations:** 1 C.D. Technologies Ltd., Beer Sheba 8445914, Israel; 2 Efi Arazi School of Computer Science, Interdisciplinary Center, Herzliya 4673304, Israel; 3 Department of Science, Technology and Society, Bar Ilan University, Ramat Gan 5290002, Israel

**Keywords:** gene sequence, gene structuredness, information function, information theory, normalized mutual information

## Abstract

We provide an algorithm for the construction and analysis of autocorrelation (information) functions of gene nucleotide sequences. As a measure of correlation between discrete random variables, we use normalized mutual information. The information functions are indicative of the degree of structuredness of gene sequences. We construct the information functions for selected gene sequences. We find a significant difference between information functions of genes of different types. We hypothesize that the features of information functions of gene nucleotide sequences are related to phenotypes of these genes.

## Introduction

The problem of analyzing symbolic sequences appears in many areas of research, such as “big data” [1] and “dynamic systems” [2]. The most significant example of a symbolic sequence is nucleotide sequence. Moreover, a nucleotide sequence is an interesting and important mathematical object. Of special importance is the task of clustering nucleotide sequences [3-6]. A nucleotide sequence is hereby referred to as a sequence whose elements assume the values A, C, G, T. First the mathematical analysis of nucleotide sequences was suggested by the physicist Gamow in 1954 [7]. The problem of symbols relation of nucleotide sequences was first discussed by the physicist Yockey in the 1950s [8]. About 50 years later, in 2003, the mathematician Gelfand noted that “the use of mathematics in studying gene sequences is an adequate language” [9]. This implied the finding of formal (mathematical) properties of gene nucleotide sequences. Yet, insufficient attention has been paid to this subject.

The main method of investigating numeric sequences (or discrete numerical time series) is the construction and analysis of autocorrelation functions. However, the principal difference of numeric sequences from nucleotide sequences is that the nucleotides in the sequence take the symbolic values A, C, G, T. This means that statistical apparatus cannot be used for the analysis of such sequences, insofar as statistics does not have theoretically justified measures of correlation between symbolic (discrete) random variables. The impossibility of utilizing theoretically justified statistical methods in genetics has been noted earlier [10]. Therefore, information theory, having a solid theoretical justification, has been increasingly used in the study of biological data. Earlier we have applied information theory to analyze data on aging-related diseases [11,12], including cancer [13-15]. The approach described in [13] is presented in the monograph [16].

An overview of the use of information theory for the analysis of biological sequences, in particular DNA sequences, has been presented earlier [17-19]. In the work by Li (1990) [20], mutual information was first used as a measure of correlation for autocorrelation symbolic sequence function. However, mutual information is a non-normalized value, and therefore it does not allow the researchers, in the general case, to compare different mutual information functions for different symbolic sequences.

The present work, for the first time, uses normalized mutual information as a measure of correlation to construct an autocorrelation function for the symbolic (nucleotide) sequence. Hence, we will refer to this function as information function. The use of normalized mutual information allows us to compare information functions of any symbolic sequences. The present article presents an algorithm for distributing sets of genes according to their information functions, that is, according to the interconnection between elements in the nucleotide sequences of these genes. Each value of the information function estimates the interconnection between elements of a nucleotide sequence with a corresponding lag. The set of all the values of the information function provides an estimate for the interconnection of the elements in a nucleotide sequence with all the lags, that is to say, it provides an estimate of the degree of structuredness of that sequence.

It may be hypothesized that genes with “close” information functions may produce similar phenotypes, and the proposed approach may help reveal unknown phenotypic properties of genes according to their nucleotide sequences.

## Methods

### Gene sequences

To illustrate the algorithm of distribution, we consider the nucleotide sequences of 14 genes. Table 1 lists the genes and their sizes as the number of nucleotides. The data on the genes’ sequences were obtained from NCBI Gene database (https://www.ncbi.nlm.nih.gov/gene).

The genes used in this study—BCL2, mTOR, FOXO3, FOXO1, IGF1, BRCA2, BRCA1, Klotho, Sirtuin 1, p16, BECN1, CCND1, Sirtuin 6, APOE—were selected for the most part insofar as these genes are often recognized as being involved in aging processes and often constitute networks in aging-associated pathways [21,22]. Hence both their phenotypic properties and their possible mutual relation could be suggested.

### Mathematical analysis

Let X be a symbolic random variable with a distribution function as follows:

$$X:\;x_{1},\;x_{2},\;\ldots ,\;x_{n}\;P:\;\;p_{1},\;p_{2},\;\ldots ,\;p_{n}.$$

Entropy of random variable X is as follows:

$$H(X)=-\sum_{i=1}^{n}p_{i}\log\;p_{i}.$$

Let X and Y be symbolic random variables. The mutual information between the variables X and Y is as follows:

I(X;Y) = H(X) + H(Y) ‒ H(XY),  

where *H(XY)* is the entropy of the product of the random variables X and Y.

Let X and Y be symbolic random variables. The normalized mutual information (also termed “uncertainty coefficient”) is as follows:

$$C(X,Y)=\frac{I(X;Y)}{H(Y)}=\frac{H(X)+H(Y)-H(XY)}{H(Y)}$$

The normalized mutual information has the following properties.

(1) *0 ≤ C(X;Y) ≤ 1*.

(2) *C(X;Y)* = *0* if and only if the random variables X and Y are independent (no correlation between the variables).

(3) *C(X;Y)* = *1* if and only if there is a functional relation (correlation or influence) between X and Y.

Let *x(n) = (x(1), x(2), …, x(n),…)* represent discrete time series having symbolic values.

Let *x(n+j) = (x(1 + j), x(2 + j),…, x(n + j),…)* be a time series x(n) with a lagj.

The auto-mutual information of the time series *x(n)* with a lagj equals:

*I(x(n); x(n + j)) = H(x(n)) + H(x (n + j)) – H(x(n), x(n + j))*.

The normalized auto-mutual information of the time series *x(n)* with a lag*j* equals:

$$C(x(n);x(n+j))=\frac{I(x(n);x(n+j))}{H(x(n+j))}=\frac{H(x(n))+H(x(n+j))-H(x(n),\;x(n+j))}{H(x(n+j))}$$

The normalized mutual information *C(x(n); x(n+j))* is then calculated as a function of the lag*j*.

We shall refer to function *F(j)=C(x(n); x(n + j))* as the information function of the discrete time series *x(n)*.

Properties of the information function F(j) are as follows.

(1) *0 ≤ F(j) ≤ 1*.

(2) *F(j)* = 0 if and only if *x(n)* and *x(n + j)* are mutually independent.

(3) *F(j)* = 1 if and only if there exists a functional relationship between *x(n)* and *x(n + 1)*.

Let *{x1(n), x2(n), …, xk(n)}* be a set of discrete time series, whose elements are symbols, e.g. gene nucleotide sequences, *n = 1, 2, 3,…*, and the maximum value *n* for a sequence *xi(n) 1 ≤ i ≤ k* equals the number of elements in this nucleotide sequence.

The algorithm of distributing a set of time series *{x1(n), x2(n), …, xk(n)}* consists of three procedures: (1) construction of an information function matrix; (2) ranking of columns of the information function matrix; and (3) application of a multiple comparisons method.

#### Construction of an information function matrix

For each time series *xi (n) 1 ≤ i≤ k*, we construct the information function as follows:

Fi(j) *1 ≤ i ≤ k, 1 ≤ j ≤ m*,

where m is the number of lags in the information function.

We obtain the *k x m [Fi(j)]* matrix of values of the information functions, i.e., a matrix where each row is an information function of the corresponding time series.

#### Ranking of columns of the information function matrix

Each row of *[Fi(j)]* matrix is an information function of time series, and each column contains the values of information functions corresponding to the same lag.

For each column of *[Fi(j)]* matrix, we rank its entries and assign the rank 1 to the smallest entry of the column. We obtain *k x m* matrix of ranks *[ri(j)]*, with each column of the matrix containing ranks from 1 to *k*.

*We estimate the element interconnection of the i-th time series as compared to the element interconnection of other time series by the sum of all the elements of i-th row of the matrix [ri(j)]*. Such an estimation allows us to use multiple comparisons of rank statistics for the comparison of time series interconnection.

#### Application of a multiple comparisons method

We compare rank sums using the Newman-Keuls test [23]. This test provides adequate results in the analysis of biomedical data, including aging-related multimorbidity [11,12], and is appropriate for the present problem.

## Results

### The values and clustering of gene information functions

Following the above algorithm for distributing a set of time series, we calculate and cluster the values of gene information functions, as follows.

(1) For each gene, out of the 14 genes under consideration, we calculate the information function with 12 lags. We obtain the information functions matrix *[Fi(j)] 1 ≤ i ≤ 14, 1 ≤ j ≤ 12* (Table 2).

(2) We rank the entries of each column of the information function matrix, while attributing to the least values the rank 1. We obtain the rank matrix *[ri(j)] 1 ≤ i ≤ 14, 1 ≤ j ≤ 12* (Table 3).

Let us consider Table 3 as the Friedman statistical model [24] and examine the row effect of this table.

*Hypotheses*:

H0: There is no row effect (“null hypothesis”).

H1: The null hypothesis is invalid.

*Critical range*:

The sample is “large”, therefore, the critical range is the upper 1%-range of χ^2^_13_ distribution.

Let us calculate the χ^2^-criterion. This gives us χ^2^ = 91.65. The critical range is χ^2^_13_ > 27.69. Since 91.65 > 27.69, the null hypothesis with respect to Table 3 is rejected. Thus, according to the Friedman test, the row effect has been found. Hence, there is a difference between the rows under consideration.

For multiple comparisons, we use the Newman-Keuls test. We obtain */R_i_ - R_i+1_/* > 8.93, where *R_i_* and *R_i+1_* are elements of the column “Sum of ranks” in the *i*-th and (*i+1*)-th rows of Table 3, respectively. By multiple comparisons, we construct the clustering shown in Table 4.

The obtained clustering possesses the following properties: (1) For two neighboring sets of Table 4, the smallest element of one set and the greatest element of another set located nearby are significantly different (*α_T_* = 0.01); (2) Elements belonging to the same set do not differ from each other (*α_T_* = 0.01).

Note that the differences between cluster 1 (APOE gene) and all the other elements (genes) are statistically significant (*α_T_* = 0.01). The same holds true for cluster 3 (BECN1 gene), cluster 4 (mTOR gene), and cluster 7 (IGF1 gene).

### The significance of gene information functions

The domain of the information functions under consideration is the set {Lag 1, Lag 2, Lag 3, … , Lag 12}, and the values are the set of real numbers 0 to 1. We perform the comparative analysis of the values of information functions on the domain of those functions.

In Table 2, each row represents the values of the information function of a corresponding gene. We rank the values of each row of Table 2, attributing rank 1 to the least value. We obtain Table 5.

We evaluate the values of the information functions in *Lag j* as the sum of elements of the column *Lag j* of Table 5. Let us consider Table 5 as the Friedman statistical model, and examine the column effect of this table.

*Hypotheses*:

H0: There is no column effect (“null hypothesis”).

H1: The null hypothesis is invalid.

*Critical range*:

The sample is “large”, therefore, the critical range is the upper 1%‒range of χ^2^_11_ distribution.

Let us calculate the χ^2^-criterion. This gives us χ2 = 121.5. The critical range is χ^2^_11_ > 24.73. Since 121.5 > 24.73, the null hypothesis with respect to Table 4 is rejected. Thus, according to the Friedman test, the column effect has been found. Hence, there is a difference between the columns under consideration.

For multiple comparisons, we use the Newman-Keuls test. We obtain */R_i_ ‒ R_i+1_/* > 9.64, where *R_i_* and *R_i+1_* are elements of the column “Sum of ranks” in the *i*-th and (*I + 1*)-th rows of Table 5, respectively. By multiple comparisons, we construct the clustering shown in Table 6.

The obtained clustering possesses the following properties: (1) For two neighboring sets of Table 5, the smallest element of one set and the greatest element of another set located nearby are significantly different (*α_T_* = 0.01); (2) Elements belonging to the same set do not differ from each other (*α_T_* = 0.01).

Note that the differences between cluster 1 (Lag 1) and all the elements are statistically significant (*α_T_* = 0.01). The same holds true for cluster 2 (Lag 2), cluster 3 (Lag 6), and cluster 4 (Lag 3).

The values of the information functions in Lag 6 are greater than the values of the information functions in Lag 3, Lag 4, and Lag 5. This signifies that, for the group of genes under consideration, the interconnection between elements distanced five elements from each other is greater than the interconnection between elements located closer together, namely distanced 2, 3, and 4 elements from each other.

## Discussion

In this work we established a novel information theory based method for the evaluation of the level of structuredness of gene sequences (information function) by the sequences’ normalized mutual information. This new method may serve as an additional structural evaluation tool for genomic analysis, and for omics biomarkers analysis generally. In the future, it may be possible to associate between the gene structuredness as evaluated by the present method and the expression and phenotype of particular genes under consideration. Here we, for the first time, describe the methodology to calculate the gene structuredness, while the association of the gene structuredness with gene expression and phenotypic function will be the task of future work.

Even though the present work only describes the methodology, some hypotheses may be advanced considering the possible association of the value of gene structuredness as shown here by the clustering (Table 4) with some known phenotypic properties of the selected genes considered in this study. Thus the genes APOE, BECN1, mTOR, and IGF1 each form a separate cluster according to their level of structuredness. This may indicate that each of these genes possesses properties not common for the other genes. The genes FOXO1 and FOXO3 are in the same cluster, which may be expected for the genes of the same group. Interestingly, the genes BRCA1 and BRCA2 are found in different clusters. As it has been demonstrated, the BRCA1 and BRCA2 genes are associated with different types of tumors, and this distinction may have been reflected in the information function (structuredness) of these genes [25,26].

Of special interest are clusters 2 and 5. Сluster 2 includes the genes Sirtuin 1 and Sirtuin 6, together with the genes CCND1, p16, and BRCA1. A special characteristic of all these five genes in cluster 2 is that under conditions of overexpression, these genes are associated with oncological diseases, though not necessarily under conditions of normal expression or under-expression [27-32]. On the other hand, a characteristic feature of cluster 5 is that all the three genes in this cluster—Klotho, BRCA2, and BCL2—under conditions of under-expression are associated with oncological diseases [33-35]. Yet, under normal expression or overexpression, such an association is not observed. Thus it may be hypothesized that the level of gene sequence structuredness, at least in the present gene selection, may be somehow associated with effects of extreme gene expression, either overexpression or under-expression. Yet, a clarification of such a hypothesis, as well as positing and testing additional hypotheses for a potential association of gene structure and function, will require further extensive investigation.

## Figures and Tables

**Table 1.** Genes used for the construction of information functions and their sizes as the number of nucleotides

No.	Gene	Gene size (No. of nucleotides)
1	BCL2	196,935
2	mTOR	166,967
3	FOXO3	124,947
4	FOXO1	110,934
5	IGF1	85,980
6	BRCA2	84,193
7	BRCA1	81,189
8	Klotho	50,083
9	Sirtuin 1	33,722
10	p16	27,292
11	BECN1	14,185
12	CCND1	13,370
13	Sirtuin 6	8,496
14	APOE	3,647

Note: In the first four genes, the numbers of nucleotides exceed 100,000.

**Table 2.** Normalized mutual information

Gene	Lag 1	Lag 2	Lag 3	Lag 4	Lag 5	Lag 6	Lag 7	Lag 8	Lag 9	Lag 10	Lag 11	Lag 12
APOE	0.027149	0.002646	0.00367	0.002773	0.003221	0.010847	0.003922	0.00447	0.004731	0.004429	0.004188	0.003196
BRCA1	0.022034	0.004159	0.003791	0.002832	0.002345	0.004025	0.00123	0.00345	0.002544	0.002233	0.002244	0.002206
p16	0.011972	0.007661	0.003112	0.003958	0.001811	0.004812	0.002174	0.003145	0.00315	0.003304	0.001438	0.002992
Sirtuin 6	0.033637	0.00474	0.002757	0.001858	0.002086	0.006216	0.001017	0.003024	0.002727	0.002613	0.000491	0.002431
Sirtuin 1	0.016229	0.004515	0.003718	0.002581	0.002479	0.003866	0.001941	0.002843	0.003234	0.001934	0.002365	0.002714
mTOR	0.025724	0.003696	0.002441	0.001687	0.001643	0.002398	0.001071	0.002092	0.001761	0.001208	0.000958	0.001191
BCL2	0.024063	0.004531	0.001164	0.002247	0.00083	0.002089	0.000997	0.001539	0.000749	0.00125	0.000493	0.001013
BECN1	0.025643	0.003635	0.00203	0.001389	0.001729	0.002412	0.001329	0.001668	0.002501	0.002151	0.001305	0.001334
BRCA2	0.019161	0.003686	0.001769	0.001204	0.001096	0.001903	0.001236	0.001632	0.0019	0.001326	0.00089	0.001334
CCND1	0.021874	0.006757	0.004128	0.003507	0.00188	0.004093	0.002921	0.001539	0.002939	0.002369	0.002	0.003324
FOXO1	0.021496	0.003354	0.001571	0.001381	0.001337	0.001946	0.000961	0.001452	0.001385	0.001303	0.00074	0.001029
FOXO3	0.023727	0.004032	0.00149	0.001242	0.001189	0.001845	0.001215	0.001161	0.00133	0.001025	0.000623	0.00093
IGF1	0.026729	0.003	0.000673	0.001228	0.000587	0.000979	0.000544	0.001135	0.000639	0.00051	0.000284	0.00048
Klotho	0.020122	0.00376	0.001905	0.001739	0.001148	0.001732	0.001059	0.001482	0.001822	0.001409	0.001414	0.001559

**Table 3.** Table of ranks: ranking by columns

Gene	Lag 1	Lag 2	Lag 3	Lag 4	Lag 5	Lag 6	Lag 7	Lag 8	Lag 9	Lag 10	Lag 11	Lag 12	Sum of ranks
APOE	13	1	11	11	14	14	14	14	14	14	14	13	147
BRCA1	7	9	13	12	12	10	8	13	9	10	12	9	124
p16	1	14	10	14	9	12	12	12	12	13	10	12	131
Sirtuin 6	14	12	9	8	11	13	4	11	10	12	2	10	116
Sirtuin 1	2	10	12	10	13	9	11	10	13	8	13	11	122
mTOR	11	6	8	6	7	7	6	9	5	3	7	5	80
BCL2	9	11	2	9	2	6	3	5.5	2	4	3	3	59.5
BECN1	10	4	7	5	8	8	10	8	8	9	8	6.5	91.5
BRCA2	3	5	5	1	3	4	9	7	7	6	6	6.5	62.5
CCND1	6	13	14	13	10	11	13	5.5	11	11	11	14	132.5
FOXO1	5	3	4	4	6	5	2	3	4	5	5	4	50
FOXO3	8	8	3	3	5	3	7	2	3	2	4	2	50
IGF1	12	2	1	2	1	1	1	1	1	1	1	1	25
Klotho	4	7	6	7	4	2	5	4	6	7	9	8	69

**Table 4.** Gene distribution according to sums of ranks

No.	Cluster	Sub-cluster	Gene	Sum of ranks
1	Cluster 1		APOE	147
2	Cluster 2	2.1	CCND1	132.5
3		2.2	p16	131
4		2.3	BRCA1	124
5		2.4	Sirtuin 1	122
6		2.5	Sirtuin 6	116
7	Cluster 3		BECN1	91.5
8	Cluster 4		mTOR	80
9	Cluster 5	5.1	Klotho	69
10		5.2	BRCA2	62.5
11		5.3	BCL2	59.5
12	Cluster 6	6.1	FOXO1	50
13		6.2	FOXO3	50
14	Cluster 7		IGF1	25

**Table 5.** Table of ranks: ranking by rows

Gene	Lag 1	Lag 2	Lag 3	Lag 4	Lag 5	Lag 6	Lag 7	Lag 8	Lag 9	Lag 10	Lag 11	Lag 12
APOE	12	11	9	7	5	10	1	8	6	3	4	2
BRCA1	12	11	5	9	2	10	3	6	7	8	1	4
p16	12	10	8	3	4	11	2	9	7	6	1	5
Sirtuin 6	12	11	9	5	4	10	2	7	8	1	3	6
Sirtuin 1	12	11	10	6	5	9	2	8	7	4	1	3
mTOR	12	11	6	10	3	9	4	8	2	7	1	5
BCL2	12	11	7	4	6	9	2	5	10	8	1	3
BECN1	12	11	8	3	2	10	4	7	9	5	1	6
BRCA2	12	11	10	8	2	9	5	1	6	4	3	7
CCND1	12	11	9	6	5	10	2	8	7	4	1	3
FOXO1	12	11	9	7	5	10	6	4	8	3	1	2
FOXO3	12	11	7	10	5	8	4	9	6	3	1	2
IGF1	12	11	10	8	2	7	1	5	9	3	4	6
Klotho	12	11	10	8	2	7	1	5	9	3	4	6
Sum of ranks	168	153	117	94	52	129	39	90	101	62	27	60

**Table 6.** Distribution of lags

No.	Cluster	Sub-cluster	Lag	Sum of ranks
1	Cluster 1		Lag 1	168
2	Cluster 2		Lag 2	153
3	Cluster 3		Lag 6	129
4	Cluster 4		Lag 3	117
5	Cluster 5	5.1	Lag 9	101
6		5.2	Lag 4	94
7		5.3	Lag 8	90
8	Cluster 6	6.1	Lag 10	62
9		6.2	Lag 12	60
10		6.3	Lag 5	52
11	Cluster 7		Lag 7	39
12	Cluster 8		Lag 11	27
